# Networking expertise: Discursive coalitions and collaborative networks of experts in a public creationism controversy in the UK

**DOI:** 10.1177/0963662510383385

**Published:** 2012-04

**Authors:** Joachim Allgaier

**Keywords:** citizens, coalitions, creationism, ethno-epistemic assemblages, experts, newspapers, public engagement

## Abstract

Experts do play a particular role in public socio-scientific debates, even more so if they form heterogeneous coalition with other actors and experts. A case study about a public science education controversy surrounding the teaching of evolution/creationism in the UK press is used to investigate in detail how connections and coalitions between experts and other actors involved in the controversy emerged and played out. The research focuses on the question of what role collaborative and other networks of experts played in terms of influence, visibility, credibility, consensus and weight of argument. Issues that are considered in the research are the status of the members of the coalitions forming during the debate and how it is displayed in media representations and letters and petitions, and also how these networks and coalitions of experts perform in relation to each other.

## 1. Introduction

Who has and who does not have relevant expertise in public controversies about science and technology has been and still is a hot topic in science studies (e.g. Collins and Evans, 2002, 2007). However, many of the public debates about scientific and technological issues concern more than just technical details and scientific expert knowledge. In many cases the key issues are framed in scientific terms (Rayner, 2003) and therefore the authority and opinions of scientific and technical experts become particularly important key resources in public controversies. Statements of scientific experts are even more credible and valuable when there is a consensus among them; consequently things get messier when the scientific and technical experts disagree in public (Thomas, 2009). In complex socio-scientific controversies it is often difficult to determine what kind of and which experts are actually seen as the relevant experts for the issues at stake, as well as who should decide who the relevant experts are (e.g. Jasanoff, 2003). Even though it is commonly understood that more than just scientific and technical expertise is relevant for solving societal problems about science and technology it seems fair to say that scientific experts enjoy a particular status as public experts (e.g. Peters, 2008). Scientific and other forms of expertise are used in different ways by opposing parties in public controversies where expertise meets counter-expertise – it is no longer the case that scientific advice is a resource that is only available to government officials or other particularly well-equipped players (e.g. Maasen and Weingart, 2005).

However, less attention has been paid to the issue of how different kinds of experts connect and collaborate in public controversies and what resources they mobilize in order to achieve credibility. In this article I am going to illustrate a case about a school in England that was accused of teaching creationism in science classes. Various heterogeneous groups of actors formed that were either attacking or defending the school. Irwin and Michael’s (2003) concept of ethno-epistemic assemblages will be used to identify discursive connections and collaboratively acting networks between the experts involved in the debate and their role in the controversy.

## 2. Networked expertise and ethno-epistemic assemblages

From a sociological and a science and technology studies perspective the idea that there is clear distinction between expert knowledge and non-expert (or lay) knowledge has been criticized and abandoned (e.g. Irwin and Wynne, 1996). A range of case studies has found that “ordinary” citizens possess situated and local knowledge(s) that is often crucial to apply abstract scientific knowledge to particular contexts and cases. It is also a valuable and significant resource in reaching decisions about public policy issues (e.g. Fischer, 2003; Corburn, 2005). Furthermore, local knowledge usefully contributes to and can become an integral part of scientific knowledge.

Rather little is known about the basis of the credibility of expertise. To investigate this matter further, Limoges (1993) offers a processual understanding of expertise in controversy contexts. Limoges describes controversies as “controversist spaces” in which various actors and experts with completely different “worlds of relevance” meet. For Limoges, all participating groups are fully fledged actors in this space; thus expertise per se does not count more than the view of any of the other involved actors, and in most cases expertise is provided in plural and often contradictory ways. Limoges asserts that the actual issue during a controversy is the negotiation of the associations established between the different “worlds of relevance” mobilized by different participants. Such associations are not defined a priori but emerge as outcomes of the interactions between the participants. Nowotny (2003: 154) asserts that “the challenges posed by the social distribution of expertise and its ongoing societal contextualization, lies in the nature and robustness of links it can build with other types of knowledge, other kinds of experience and expertise.” In other words, the robustness, representation and credibility of expertise develop in the course of a particular controversy. How powerful and credible experts become in a particular controversy depends in this view on their ability to build networks and form associations with the other actors involved in the controversy.

The credibility of expertise needs to be developed within a controversy context and it is therefore not an individual property but a process of collective attribution. For Limoges the credibility of expertise in public controversies stems from the strengths of the networks with which experts are associated in the controversy. Expertise, in this view, is therefore seen as a collective learning process, which provides the experts with positive appraisal if they are successful in addressing the articulations of various “worlds of relevance.” In this sense, expertise is a public process, which creates the conditions of credibility of expert performance (Limoges, 1993).

This notion of a networked and collective form of expertise in public controversy contexts points to formations of heterogeneous alliances between different kinds of experts and citizens. Irwin and Michael (2003) propose the concept of ethno-epistemic assemblages that transcend the boundaries between experts and laypersons in order to analytically grasp such new forms of interweavings between science, technology and society, and people, texts, discourses and objects. Irwin and Michael suggest that in practice there is less a contrast between expert and lay actors in issues concerning science and citizens but that it is rather coalitions or assemblages of various actors that emerge and face each other. Here, they draw specifically on the work of Deleuze and Guattari (2004) on assemblages. The term “assemblage” connotes an anti-structural concept that stresses emergence, heterogeneity and the decentred-ness of the order of social life (Marcus and Saka, 2006). In scientific and technological issues that concern various actors one finds different coalitions that bring together laypeople and experts in the same group. These assemblages can entail expert practitioners such as communication or publicity experts but also experts with knowledge from different areas such as political, scientific, religious expertise and local, experiential and other types of knowledge.

Irwin and Michael (2003) argue that in practice it is such assemblages that are in conflict rather than experts and laypeople or science and society per se. These ethno-epistemic assemblages battle with one another for legitimacy and credibility in the wider public and also often try to gain influence on decision-making processes and use the media to do so. “Epistemic” here refers to the production of truth or truth claims; “ethno” connotes the idea of locality and situated-ness of knowledge; and the concept of “assemblage” is used to grasp interweavings of lays and experts (Irwin and Michael, 2003: 119–20). These assemblages are not static, they are dynamic and processual, and different actors with various knowledges, expertise and experience can join such groups, but also leave and abandon them if they are not successful. This concept is proposed for a better understanding of the way controversy, debate and negotiation are actually played out in public.

## 3. The case of Emmanuel College

A public debate about teaching evolution/creationism in the UK brought together experts from science and education, religious authorities, high-profile politicians and spokespeople of government departments, representatives of atheist, humanist and religious non-government organizations (NGOs) and action groups, creationist organizations, media professionals and commentators as well as teachers, parents and pupils. The range of issues addressed reached, among others, from new types of schools and sponsorship in education, to the epistemological status of the theory of evolution, the nature of science and purposes of (science) education, issues of free speech and free choice and the role of religion and faith in multicultural and multi-faith societies (Allgaier, 2008).

In January 2002, it was reported in a newspaper specializing in education coverage that educators at Emmanuel College in the north of England rented rooms to Answers in Genesis, a creationist organization that was going to hold a conference at the school in March (Dean 2002a). On the weekend the conference was held, a series of articles in the quality newspaper *The Guardian* accused the school of teaching creationism in science classes and the educators of undermining the theory of evolution (e.g. Branigan, 2002).

Emmanuel College was not just any school – it consistently received excellent exam results. The Office for Standards in Education (OFSTED) wrote a very favourable report about the school and the government designated it the status *beacon school*. Another issue that was central in the debate was that the school, as a city technology college, was partly funded by the state and partly also by the private sector. The sponsor of the school was Sir Peter Vardy’s Vardy Foundation, a charitable trust with a Christian ethos (it was later renamed the Emmanuel School Foundation as a consequence of the press coverage). A member of the political opposition picked up the issue of Emmanuel College being reported as teaching creationism in science classrooms in Parliament and asked the Prime Minister, Tony Blair, about his views on the issue. Tony Blair backed the school for its good exam results, considered that the newspaper reports were exaggerated and said that he welcomed diversity in education (e.g. Kallenbach, 2002). The educators and the sponsor of Emmanuel College denied the accusations and stressed that all requirements of the National Curriculum were followed in the college – which was later confirmed by the relevant education authorities. Emmanuel College spokespeople emphasized the openness of the college to children of various cultural backgrounds and that at the school the children were offered both, religious views on creation and the theory of evolution, so that children could decide for themselves what they wanted to believe.

The issue of creationism being taught in science classes at Emmanuel College was reported in many newspapers – and it was also reported in scientific journals (e.g. Gross, 2002) and other media outlets (e.g. TV and radio news). Various action groups got together in the course of the controversy to write petitions and call for action concerning the controversy. Opponents of the school consistently claimed that religious “indoctrination” and “brainwashing” would take place and that the educators of the school and the Christian sponsor were “peddling” creationism in the school and undermining the teaching of Darwin’s theory of evolution (Allgaier and Holliman, 2006).

## 4. Methods

This contribution is part of a more comprehensive study on the public representation of science education (Allgaier, 2008). In this original investigation, 287 newspaper articles were sampled from eight national UK newspapers and their Sunday equivalents. Also included in this sample, were two regional/local newspapers (from the geographical area where Emmanuel College is situated) and two UK newspapers specializing in education reporting.^1^ These sources were analysed using qualitative and quantitative approaches to content analyses.

The news reports were sampled starting 1 January 2002 and ending on 20 February 2004. The newspaper content was retrieved from the electronic newspaper archive LexisNexis. All the articles referred to the debate around Emmanuel College during that time. They were coded for distribution over time and newspapers, names and bylines of the correspondents, types of articles, number of directly quoted sources, as well as the different issues of the controversy that were reported in the course of the debate.^2^

In order to investigate the distribution of types of experts and other sources a quantitative approach of analysing media content was adopted and six categories of sources, based on their description in newspaper reports, were set up.

- scientific experts and institutions;
- educational experts and institutions;
- NGOs, campaigners and action groups;
- politicians, authorities and other officials;
- religious experts and institutions;
- media professionals and organizations;

Additionally, a seventh category for parents and/or pupils was included, following the methodological recommendations of Limoges (1993). This analysis investigated the distribution of directly quoted sources in newspaper articles but not in letters (because they are written by the readers of newspapers). The analysis is therefore limited to expert and other source representation in newspapers. Furthermore, a qualitative approach was adopted that examined and categorized all statements from sources in quotation marks in all articles, apart from the letters. The same seven categories were used in this analysis in order to be able to make consistent comparisons between the findings of the different types of analyses. Similarities and differences in the arguments of the expert sources of the same category were investigated, using open, selective and axial coding procedures (see Flick, 2006). Here, it was of interest if the quoted experts of the same category all addressed the same or different issues of the controversy and if they used similar or different types of arguments in addressing the various issues of the controversy.

Furthermore, the newspaper articles were checked systematically in terms of connections between the quoted expert and other sources. All types of articles were included in this process, including the letters because some of the experts wrote letters themselves or were co-signatories in commonly drafted letters. A selective coding strategy (Flick, 2006) was therefore employed to filter out all the sequences referring to connections between expert sources. The resulting categorization was based on what the actors were arguing for, what kinds of issues their arguments concerned and what common aims the experts and actors had.

As an additional data collection strategy, online searches were conducted to check what information was available on the web. Search engines such as Google and the searchable online archives of newspapers and other media outlets were used to look for connections between the expert sources, institutions and organizations represented in the debate, as well as for relevant documents, such as letters, petitions and reports. As a result an information archive was created that collected all materials obtained through the internet that supplemented the newspaper sample.

## 5. Findings: Experts, discursive coalitions and collaborative networks of experts

A total of 304 expert and other sources were quoted directly in the sample. To find out more about the role of experts in a public controversy it is important to know not only how many and what kinds of experts are quoted in the reporting, but also what they are actually saying concerning in the disputes and which of the various controversial issues they address.

### Education experts

Education experts were quoted 81 times. The education experts of Emmanuel College (53 quotes), foremost principal Nigel McQuoid (17 quotes), consistently defended the teaching practice of the school by pointing to the good results that the school achieved. They also said that their teaching practice met the legal requirements of the National Curriculum. The main argument for teaching creationism and the theory of evolution was the recourse to an informed choice argument; i.e. they said they were offering both versions to the children so that they could make up their minds and decide for themselves what to believe.

The education experts related to teaching unions (10 quotes) criticized Emmanuel College but their main argument was against sponsorship in public education. Here it was claimed that Vardy sponsored the school so that he could influence what is taught at the school and claims about religious “indoctrination” at the school were made.

Various further individual education experts (18 quotes) addressed various issues of the debate and had varying opinions on these.

### Scientific experts

Scientific experts were quoted 76 times. The group of scientific experts was dominated by Richard Dawkins (21 quotes – the source quoted most often in the sample) and other scientists that underlined the superior epistemological status of the theory of evolution referring to empirical evidence and attacked the validity of creationist theories for not being scientific theories (together 69 quotes). For these scientific experts evolution and religious belief were incompatible and the boundaries between religion and science needed to be defended in science education. In this regard, these scientific experts used the debate around teaching creationism (and particularly arguments about the content of the science curriculum) as a site for boundary work (see Gieryn, 1983, 1995, 1999), defending the boundaries between science and non-science.

However, three experts in this category attacked the epistemological status of the theory of evolution and challenged the teaching of evolution in science education, arguing that the scientific base for evolution was weak. These scientific experts wanted to use the science curriculum to challenge the consensus view of the scientific community on evolution.

Also, four of the quoted scientific experts argued that scientific and religious accounts did not contradict each other and that both could be true. Subsequently, they argued that there is no conflict between scientific and religious accounts, but that the two address different types and sets of questions. Consequently, none of these four scientific experts challenged the epistemological status of the theory of evolution or the teaching of evolution in science classes.

### NGOs and action groups

The NGO, action group and campaigner category had 56 quotes. Secular and humanist organizations and a parent action group argued against the influence of sponsors in education and used the story of Emmanuel College teaching creationism in science classes as an example for the abuse of influence through sponsors in state education (together 21 quotes). Here it was claimed, similar to the case of the teaching unions, that Peter Vardy sponsored the school in order to “peddle” creationism at the school and to “brainwash” children.

Peter Vardy and the Vardy Foundation (together 19 quotes) consistently denied these claims and said that sponsorship of the school was a result of him feeling responsible for the community he lived in. Vardy and the Vardy Foundation, similar to Emmanuel College educators, also pointed to the good results of the school and used the informed choice argument; i.e. that children are taught both theories so that they can make up their minds.

Creationist organizations (14 quotes) that were quoted in the coverage addressed a different issue: the moral dimension of the belief in evolution. For them the theory of evolution was more than a scientific theory. They saw it as a materialist/atheist worldview that denied the purpose of human existence and therefore led to sin. The issue in the quotes of this category is therefore not only the question of whether creationism or the theory of evolution is “true” and what should be taught in schools, but also the issue of what is the “morally” correct view.

### Political experts and authorities

There were 40 quotes from politicians, authorities and other officials. In the group of these political experts Prime Minister Tony Blair (12 quotes) backed the school for its good results, OFSTED (four quotes) and the Department for Education and Skills (five quotes) confirmed that the school met the requirements of the National Curriculum and members of the opposition (together nine quotes) attacked the Prime Minister for backing the school and challenged the credibility of OFSTED for writing a favourable report about Emmanuel College. The remaining 10 quotes were from other individual politicians and spokespeople of authorities and concerned various further issues.

### Religion experts

Religious experts and clergy (27 quotes) also had diverse arguments. Most religious experts and clergy did not see religion and science in competition but explained that they both tried to answer entirely different questions; for instance, that sciences answer questions about *how* natural processes take place, whereas religions ask *why* these processes happen at all. Some of them criticized Emmanuel College and creationism for bringing Christianity into disrepute, few backed the school for its good results and one religious expert attacked the theory of evolution with a similar (moral) argument to that used by the creationist organizations: evolution is a “hoax” disseminated by the atheist scientific community that leads to mindless violence and sin.

### Pupils and parents

Most of the quotes in the parents and pupils category (20 quotes) were from pupils at Emmanuel College and their parents (together 16). These defended the school for its success in education, the excellent results it achieved and also used the informed choice argument in order to defend the school from the allegations that they were “brainwashing” children.

As a result, expert sources from the same expert groups addressed different controversial issues within the debate and did not have the same views on the various controversial issues of the debate. For instance, although all scientific experts in the sample were drawing on a similar rhetoric to make “scientific” claims and arguments, a minority of scientists came to very different conclusions concerning the debate around teaching the theory of evolution and creationism (Allgaier, 2010). The results lead to the conclusion that there was a complex range of personal views on the topic among individual experts and between groups of experts. However, it is the journalists who decide who is selected and how the sources are described (Allgaier, 2008). The picture of a simplistic dichotomy, for instance that one group of experts had a common view on a topic that is opposed to the consensus view of another group of experts or a lay audience, is challenged by this interpretation.

### Anti-Emmanuel College/pro-evolution coalition

The organizations, groups and individuals attacking Emmanuel College had different reasons, aims and intentions for getting involved in this particular public debate. Also the issues that they addressed were different ones and ranged from the National Curriculum to sponsorship in education, and to a general critique of governmental authorities and policies. However, following the newspaper coverage about the issues all the experts and groups had in common that they were arguing against the teaching of creationism and opposing Emmanuel College. Therefore these experts formed one discursive coalition against Emmanuel College and the Vardy Foundation that was held together by a particular story line (Hajer, 1997).

This “assemblage” (Irwin and Michael, 2003; Deleuze and Guattari, 2004) consisted of diverse elements, issues and experts. It united anti-creationist scientists (e.g. Richard Dawkins), education experts (e.g. various teaching unions and individual teachers), NGOs and campaigners (e.g. the British Humanist Association and the National Secular Society); politicians (e.g. the opposition Members of Parliament (MPs) Jenny Tonge and Phil Willis); and religious experts and clergy (e.g. Bishop of Oxford Richard Harries and other bishops) around a single issue. This heterogeneous assemblage was also backed through a variety of letters and comment writers, such as e.g. *Guardian* (2002) or Carr (2002) arguing against and undermining the expertise of the educators at Emmanuel College and the Vardy Foundation. However, within this coalition that was tied together by the arguments of the experts and their common goal to attack Emmanuel College for various reasons there were several action groups that came together to write petitions to representatives of the government about particular issues of the controversy.

Branigan and White (2002) reported that “leading scientists” called on OFSTED to re-inspect Emmanuel College because Christian teachers there did not believe in evolution and were undermining the scientific teaching of biology. Furthermore, it was reported that the appeal for re-inspection was backed by a politician: Dr Jenny Tonge of the Liberal Democrats, the MP that questioned Prime Minister Tony Blair on the issue of creationism being taught at Emmanuel College. It was also reported that “leading philosophers” had signed a British Humanist Association petition that urged the government to clarify the wording of the National Curriculum so that teachers could not present creationist theories as the scientific equivalent of evolution (Branigan and White, 2002; Clancy, 2002). Cassidy (2002) reported that a group of “secular campaigners” including scientists, philosophers and church leaders, called on ministers to ban the teaching of creationism in state-funded schools.

Dean (2002b) reported that 43 scientists and philosophers had signed a petition. It was organized by the British Humanist Association and called for legal requirements in the National Curriculum for Science to be tightened to prevent creation stories being taught as anything other than religious myths. The petition was sent to Tony Blair and copies were also sent to the Education and Skills Secretary Estelle Morris and other educational authorities. This petition was aimed explicitly at the National Curriculum for Science and argued for a change of a formulation in Key Stage 4 (Years 10 and 11 – ages 14 and 15 – in England and Wales) that refers to teaching scientific controversies arising from different ways of interpreting empirical evidence (Archard et al., 2002). It was of special concern to the members of the British Humanist Association that the theory of evolution was mentioned in brackets. This formulation made it possible for the educators at Emmanuel College to bring in alternative explanations in science education and to still fulfil the legal requirements of the National Curriculum (see also Allgaier and Holliman, 2006).

Further heterogeneous alliances appeared in the coverage: Robin McKie (2002) wrote that “an unprecedented amalgamation of the country’s top religious and scientific leaders” had called on Tony Blair to express their “growing anxiety” over the spread of faith schools in Britain. The warning of the group that was led by the Bishop of Oxford, Richard Harries, and biologist Richard Dawkins followed the news that Gateshead’s Emmanuel College had included creationism in biology lessons.

In a letter to the Prime Minister this miscellaneous group – which included various bishops and scientific experts as well as the popular TV naturalist David Attenborough – expressed concern over the introduction of creationism in British schools. This petition was signed by nine scientists (apart from one all are members of the Royal Society) and seven bishops (Harries et al., 2002). First, it is noteworthy that the outspoken atheist Richard Dawkins teamed up with high-profile churchmen. A closer examination of the petition reveals that the explanatory power and the superior epistemic status of the theory of evolution were emphasized in the letter (and no reference to any benefits of faith or religion was made). Here, the letter attacked the statement by Emmanuel College sources that the theory of evolution is just a “faith position” like the belief in the creation myths in the Bible. The last section of the petition seemed to aim at the new type of school – Emmanuel City Technology College is explicitly mentioned – more generally and suggested that the teaching content in schools of this type must be monitored more carefully to avoid the blurring of the boundaries between science and religion. The text of the petition therefore also indicated that the science curriculum and media reporting are important sites for the boundary-work of science (Gieryn, 1983, 1995, 1999).

Finally some citizens in the form of concerned parents entered the scene: Brayshay (2002) and Jennings (2002) reported that a group of parents was concerned about the involvement of the Vardy Foundation in a school in their area and that they feared religious indoctrination. The parents had therefore set up an action group.

A small coalition of experts deserves special mention in this category. The view that religion and science do not contradict each other and are not necessarily in conflict was expressed in a few articles, primarily by religious experts and clergy (e.g. in Purvis, 2002), by a small minority of educators and scientists (e.g. in *Independent*, 2002); by a few letter writers and also in a few comments (e.g. Vallely, 2002).

Letters by the organizations Christians in Science (Burke et al., 2002) and the Association of Christian Teachers (Wilkins, 2002) clarified that the members of these organizations saw no conflict between the theory of evolution and their Christian belief.

The organization Christians in Science also wrote a letter to the Prime Minster. The authors stressed that the majority of Christians “have no problems with the current scientific view” and argued against a literal interpretation of the Bible. The letter was signed by 21 academics from three academic fields – science, religious education and science education.^3^ Effectively, this small sub-group defended the scientific status of the theory of evolution and rejected the teaching practice of Emmanuel College from a Christian point of view.

### Emmanuel College, the Vardy Foundation and their supporters

The educators of Emmanuel College and the Vardy Foundation also formed a heterogeneous coalition with other actors. This discursive coalition defended and supported Emmanuel College. It also had various concerns, intentions and issues and addressed the National Curriculum for Science, sponsorship of state education and the favourable report by OFSTED. Similar to the opposing assemblage attacking Emmanuel College it consisted of various elements and actors, a few scientific experts (e.g. Andy McIntosh); education experts (Emmanuel College sources); NGOs and campaigners (The Vardy Foundation, The Christian Institute, creationist organizations); politicians and authorities (Prime Minister Tony Blair, OFSTED and the Department for Education and Skills); religious experts and clergy (Bishop Michael Turnbull; Reverend Richard Harrison); and parents of pupils at Emmanuel College and the pupils themselves. This assemblage also received support through a range of letter writers and from commentators in the press (e.g. Phillips 2002; Utley, 2002). Within this coalition there was also one concrete action group that came together to write to representatives of the government to address a particular issue of the debate.

Dean (2002c) reported that a group of 30 scientists and academics had told the Education Secretary Estelle Morris that creationism and evolution should be considered side by side in school science lessons. Their letter opposed recent calls by “eminent” scientists and philosophers for changes to the National Curriculum in the wake of the row over creationism in schools. The group – which was described as including “specialists” in scientific disciplines at UK universities such as biology, physics, geology and chemistry – challenged the view that only one theory of life’s origins, namely evolution, should be taught in schools. They called for “objectivity in the curriculum.” Their spokesman Andy McIntosh (2002) also wrote a letter in which he defended Emmanuel College for its “excellent work” in debating creation and evolution and asked if Richard Dawkins’s and others’ comments were driven by science or their personal atheism. McIntosh (2002) wrote in his letter that there is “little hard experimental evidence for the evolutionary hypothesis.”

The group signing the petition said it wanted schools to teach children how to think and not what to think. Creationism should not be immediately excluded, neither should evolution. The two needed to be considered carefully and there should be an open-minded discussion in the classroom that examines the evidence (McIntosh et al., 2002).

The argument of the petition was close to the argument of Emmanuel College educators that stresses their openness and open-mindedness and relates to their argument of informed choice. The main corpus of the petition was framed in a scientific rhetoric and the importance of the “scientific method” was explicitly mentioned. The mainly scientific experts signing the letter were opposing a change to the controversial paragraph of the National Curriculum for Science at Key Stage 4 about scientific controversies, so that “alternative theories” could still be introduced in science classes alongside the theory of evolution. This petition also addressed the boundaries of science and indirectly backed the teaching practice at Emmanuel College. Maintaining the status quo would help them doing so also in the future and still be within the requirements of the National Curriculum. Having a look back at the conference held at Emmanuel College one finds that the spokesman of the group, Andy McIntosh, was also one of the speakers at the creationist conference organized by Answers in Genesis.

## 6. Discussion

Irwin and Michael’s (2003) notion of “ethno-epistemic assemblages” – a heuristic tool set up to conceptualize the complex interweavings in science–society relationships – was used to conceptualize the groups and coalitions that formed in the debate around Emmanuel College. The assemblages that appeared in the press coverage consist of discursive coalitions and collaborative networks of experts that came together to write petitions and call for action. Two ethno-epistemic assemblages could be identified in the sample: first, an assemblage attacking Emmanuel College, which includes a heterogeneous sub-group that does not see science and religion being contradictory, and, second, an assemblage defending Emmanuel College. These assemblages do cut across expert categories and contain, e.g. scientific, education and religious experts, politicians, NGOs and action groups, as well as parents and/or pupils. They employed different truth claims and worldviews (e.g. scientific arguments, political arguments, moral/ethical arguments etc. – the “epistemic” dimension) and contained different types of situated and local knowledge (from distanced observing and commenting academics to local parents and pupils that were directly affected by the results of the debate – the “ethno” dimension).

The findings also mean that in this controversy it was not the case that a group of experts of one category faced a group of experts from another category (as for instance scientific experts versus religious experts). Instead, coalitions that entailed different forms of expertise and knowledge formed to pursue common goals and faced other heterogeneous coalitions with opposing aims. In practice, one seems to encounter collective and plural forms of expertise instead or alongside struggles of single expert actors. (Online) networking with other experts can be one successful strategy to enhance credibility and to address various “worlds of relevance” (Limoges, 1993).

The experts and actors represented in the newspapers were not only passive commentators in the controversy but some of them also formed action groups in order to influence decision-making processes about some of the issues arising from the coverage about the controversy, for instance by writing a letter to a representative of the government. Here it is possible that the collection of different forms of expertise in action groups was a strategy to receive media attention and enhance credibility through the display of codified consensus. Holliman (in press) asserts that in the public sphere (scientific) consensus is hardly found in manifest forms and might only become visible when consensus is challenged and requires defending or repairing.

It is also noteworthy that written letters and petitions allow the signatories to add credibility to their names: in addition to the name, expert status of the individual signatory is further underlined by adding academic or ecclesiastical titles (e.g. Doctor; Professor, Reverend, Bishop), titles of nobility or of special national achievements (e.g. Sir, CBE; KBE^4^), honorary membership in special professional societies such as the Royal Society (FRS), The Institute of Biology (FIBiol) or the Royal College of Surgeons (FRCS), together with professional domains and affiliated institutions. For instance, one signature to the petition that was signed by bishops and scientists reads “Lord May of Oxford, President of the Royal Society” another “Sir Martin Rees, FRS, Astronomer Royal” and Andy McIntosh’s name is followed by the letters “BSc, PhD, DSc, FIMA, CMath, FInstE, CEng, FinstP,^5^ Professor of Thermodynamics and Combustion Theory, University of Leeds,” when he signed the petition initiated by him.^6^ Here it is likely that the display of status and sometimes also media prominence is used to recruit the press, and in some cases signatories to petitions have different “types” of titles, which can be seen as signifiers for status and expertise, added to their name. The message seems to be that these individuals are not just “ordinary” citizens – their expertise and status are invoked to give their argument more weight, and maybe that does also imply for some more “right” to have their views represented in the media.

The analysis illustrated that within the different categories of experts there were varying views on the controversial issues of the debate and experts, who wanted to publicly engage with the issues of the controversy, formed collaborative networks with experts from other expert fields in order to enhance their overall credibility, visibility and weight of argument. Display of status, expertise and codified consensus were central resources in the battle for publicity and influence.

Action groups demonstrated the consensus on an issue among the signatories. This consensus can be particularly credible and effective if experts from different areas are seen to agree on a position concerning a controversial issue, for instance, when scientific and religious experts together sign a petition saying that the biblical account of creation is a “faith position” and that the theory of evolution is not. Moreover, it is also possible that the (unusual) combination of different types of experts was a strategy to get access to the media.

It is probable that coalitions were formed to take action against opposing coalitions (see also Michael, 2009). The results of the study suggest that newspaper coverage of the controversy around Emmanuel College provided a platform for various individuals, interest groups and institutions, who partly used newspaper reporting (and the internet) as an important channel to argue their case (Petersen et al., 2010). The petitions written in the debates around Emmanuel College illustrate the ability of a number of interested parties to mobilize and form alliances of experts and citizens and campaign on specific issues, thereby attempting to enhance their own credibility and legitimacy and to undermine the credibility and legitimacy of opposing groups. Beck (1992) describes this mobilization of resources in controversy contexts as the politics of expertise and counter-expertise.

However, the findings of this study also point to the methodological difficulty of systematically investigating assemblages of actors and of differentiating expertise, because many of the experts were members of various social groups and it is not always clear whether their statements were based on their specialism, experience and expertise, personal opinion or a particular worldview they personally promoted. Effectively, it is the journalists’ descriptions that determine whether sources are presented as citizens, experts or spokespeople of certain groups and organizations, with or without added credibility (Allgaier, 2008, 2010).

Coalitions of experts and citizens are transient and might be formed only in controversy contexts and break up and disappear once the controversy has been resolved or when they go on for long periods without being resolved. For instance, one of the most quoted experts in the sample, the atheist scientist Richard Dawkins, who signed a petition together with various bishops in the Emmanuel College controversy, is quoted in 2007 in a newspaper article saying:

If we are too friendly to nice, decent bishops, we run the risk of buying into the fiction that there’s something virtuous about believing things because of fate rather than because of evidence. (quoted in Jha, 2007: 11).

This suggests that it is unlikely that Dawkins would like to cooperate with his former co-signatories again. It shows that particular coalitions and assemblages are formed at particular times to achieve specific goals; often they are tenuous in substance, need constant (rhetorical and practical) reproduction and are liable to fall apart at any moment. Moreover, years after the initial controversy around Emmanuel College new petitions were signed and new coalitions appeared. For instance, in October 2006 it was reported that the Emmanuel School Foundation (the former Vardy Foundation) wanted to sponsor another school in Blyth. After opponents of the school argued that creationism would be taught at the school in science classes, more than 1000 parents signed a petition against the Vardy Foundation sponsoring the school. It was reported that this petition was backed by local politicians, teaching unions and scientists (Basnett, 2006). The politicians and scientists and other “experts” that sign petitions are generally named when a petition is mentioned in news articles, the “ordinary” citizens, such as the more than 1000 parents, have to remain anonymous and seem to count in numbers only. Therefore, once again, the question arises about whose voice counts, or counts more, and on what basis these judgements are made (see also Moore and Stilgoe, 2009). In the Emmanuel College case the assemblage that managed to enrol the most diverse set of people into their network was also the most successful in achieving its goals (Allgaier and Holliman, 2006).

It is argued that the various experts involved in a controversy can form collaborative networks in order to pool resources and enhance their credibility and influence in public debates. Networking processes between expert sources could be found between various groups involved in the public debate around Emmanuel College. The issue of networks of expertise in order to enhance credibility and influence in public debates is theoretically still relatively under-examined (see Limoges, 1993; Irwin and Michael, 2003) and this study provides further empirical evidence for the importance of understanding expertise not only in individual (i.e. expert versus lay actor/expert versus counter-expert) but also in collective and networked terms.

## Notes

1. The names of the sampled newspapers are: *Daily Telegraph, Sunday Telegraph, The Times, The Sunday Times, The Guardian, The Observer, The Independent, The Independent on Sunday, Daily Mail, Mail on Sunday, Daily Express, Sunday Express, The Sun, News of the World, Daily Mirror, Sunday Mirror, The Journal* (Newcastle), *The Northern Echo* (Darlington), *Times Educational Supplement* (TES), *Times Higher Education Supplement* (THES).

2. For a more detailed account of the methods used and the sampling strategy, see Allgaier (2008).

3. The whole letter is available on the Christians in Science (CiS) website (Poole, M. W. et al. 2002) where it says that not all of the co-signatories are members of Christians in Science (CiS) and that it should not be taken as representing the view of all CiS members.

4. The Order of the British Empire awards are given in various ranks, for instance: Knight Commander (KBE) or Commander (CBE). Here the reader might be reminded of the works of Steven Shapin in which he examined the types of social figures that were participating in formal inquiry in 17th-century England: the English gentleman: “(1) the truthfulness of both the philosopher and the gentleman emerged out of the acknowledgement that these were quite special sorts of persons, and (2) for both, the virtue of the person underwrote the veracity of his narratives.” (Shapin, 1995: 398).

5. Bachelor of Science (BSc), Doctor of Philosophy (PhD), Habilitated Doctor of Science (DSc), Fellow of the Institute of Mathematics and Its Applications (FIMA), Chartered Mathematician (CMath), Fellow of the Institute of Energy (FInstE), Chartered Engineer (C.Eng), Fellow of the Institute of Physics (FInstP).

6. Another interesting signature that mixes various signifiers of status and expertise is the following example of a co-signatory of the petition affiliated with the organization Christians in Science: “Sir John Houghton, CBE, FRS, formerly Chairman of the Royal Commission on Environmental Pollution, Chief Executive of the Meteorological Office, Professor of Atmospheric Physics at the University of Oxford.”
